# Validation of blood-based transcriptomic circadian phenotyping in older adults

**DOI:** 10.1093/sleep/zsac148

**Published:** 2022-06-28

**Authors:** S Kendall Smith, Peter Tran, Katherine A Madden, Jill Boyd, Rosemary Braun, Erik S Musiek, Yo-El S Ju

**Affiliations:** Department of Anesthesiology, Washington University School of Medicine, St. Louis, MO, USA; Center on Biological Rhythms and Sleep (COBRAS), Washington University School of Medicine, St. Louis, MO, USA; Department of Neurology, Washington University School of Medicine, St. Louis, MO, USA; Department of Neurology, Washington University School of Medicine, St. Louis, MO, USA; Department of Neurology, Washington University School of Medicine, St. Louis, MO, USA; Department of Molecular Biosciences, Northwestern University, Chicago, Illinois, USA; Department of Anesthesiology, Washington University School of Medicine, St. Louis, MO, USA; Center on Biological Rhythms and Sleep (COBRAS), Washington University School of Medicine, St. Louis, MO, USA; Department of Neurology, Washington University School of Medicine, St. Louis, MO, USA; Hope Center for Neurological Disorders, Washington University School of Medicine, St. Louis, MO, USA; Department of Anesthesiology, Washington University School of Medicine, St. Louis, MO, USA; Center on Biological Rhythms and Sleep (COBRAS), Washington University School of Medicine, St. Louis, MO, USA; Department of Neurology, Washington University School of Medicine, St. Louis, MO, USA; Hope Center for Neurological Disorders, Washington University School of Medicine, St. Louis, MO, USA

Circadian rhythms govern interorgan coordination and harmonize internal function with the external environment. Age-related changes in circadian rhythms are associated with a diverse array of diseases including neurological disorders [1]. Moreover, circadian dysfunction occurs prior to symptoms in some conditions such as Alzheimer disease, suggesting a potential target for intervention [2, 3].

Current methods for circadian measurement in humans have lower granularity or are logistically constrained. Dim light melatonin onset, the “gold-standard” measure of circadian phase, requires timed dim light conditions. Actigraphy is influenced by extra-circadian behaviors and is challenging in people with limited mobility. However, newer approaches using transcriptional biomarkers may provide more granular and objective information about circadian function.

TimeSignature (TS) is an algorithm that estimates internal circadian time from gene expression in whole blood [4]. In healthy young adults, TS accuracy is maintained using as few as two suitably spaced blood samples. An additional advantage includes robust accuracy across study populations, protocols, and assay platforms [4]. However, there are no studies evaluating TS performance in older adults. In this study, we applied TS to whole-blood RNA sequencing (RNA-Seq) data from older adults and examined associations with several standard methods of circadian assessment.

All participant procedures were approved by the Washington University Human Research Protection Office. Written, informed consent was obtained from community-dwelling adults aged *>*65 years. Exclusion criteria were neurological disorders or contraindications to study procedures. Participants completed the Horne-Ostberg Morningness–Eveningness Questionnaire (MEQ) and were categorized as “morning type” (MEQ *>*59), “intermediate type” (MEQ 42–58), or “evening type” (MEQ *<*41) [5]. Participants wore actigraphs on the nondominant wrist while keeping a sleep diary, for 5–14 days at home. Then, at an overnight study visit, they provided saliva samples via passive drool method hourly from 6 pm until bedtime in a dim (<30 lux) environment. Saliva samples were immediately frozen at −20°C. Blood was collected by venipuncture at ~8 pm and ~10 am the next morning into EDTA tubes and then PAXgene RNA tubes. EDTA tubes were immediately placed on ice until centrifugation within 1 hour, followed by plasma-aliquoting and freezing/storage at −80°C. PAXgene RNA tubes were frozen per manufacturer’s protocol.

RNA was isolated using the PAXgene Blood RNA extraction kit (Qiagen), and library preparation was performed using ribosomal and globin depletion methods (Qiagen FastSelect [H/M/R+Globin]). Bulk RNA-Seq was performed on an Illumina NovaSeq S4 at 50 million reads/sample. Transcripts were processed with bcl2fastq, STAR (using Ensembl release 76), Subread, Salmon, EdgeR5, and custom Python scripts. RSeQC was used for quality control. An existing microarray data set was used for TS training before TS application as previously described [4]. The time predicted by TS is the “Transcriptomic Time.” The difference between Transcriptomic Time and True Time of sampling, or the “Transcriptomic Angle,” was calculated separately for AM and PM samples for each participant, then averaged.

For non-TS circadian assessment, saliva and plasma melatonin levels were assayed with commercial kits (Buhlmann Melatonin RIA or ELISA). Melatonin plots were visually inspected, and those with a typical “hockey stick” shape were included [6]. Mean saliva:plasma melatonin ratios were calculated to establish assay-specific thresholds equivalent to plasma melatonin levels of 10 pg/mL. DLMO was calculated by linear interpolation. Actigraphy data were processed using Actiware (Philips-Respironics) and Clocklab (Actimetrics) as previously described [3, 7, 8]. Sleep variables included those of timing (bedtime, waketime, and midsleep), quantity (total sleep time), and quality (sleep efficiency, wake time after sleep onset). Circadian variables included those of phase: M10 (indicating the start time of the most active 10 hours), amplitude, intradaily variability, and interdaily stability. Statistical analysis included tests of normality by visual inspection and Kolmogorov–Smirnov test. Student’s *t*-tests and chi-squared tests were used to compare two groups. Bland–Altman plots were generated between Transcriptomic Angle and other variables. Pearson correlations were used for associations and partial correlations for adjustment for age and sex, all in R.

**Figure 1.**
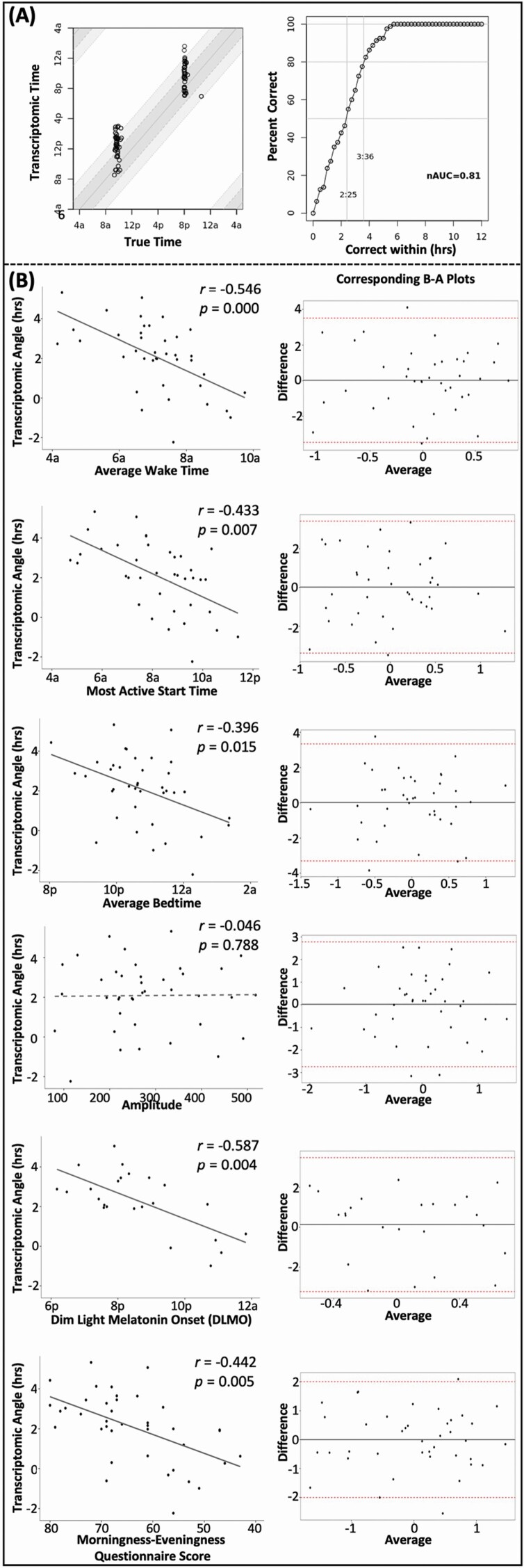
TimeSignature (TS) accuracy and relationship with standard circadian measures. (A) TS-derived Transcriptomic Time compared with the True Time of blood sampling. Dark gray bands depict Transcriptomic Angle range of *+*2 h, and light gray bands depict range of *+*4 h about the True Time. The fraction of correctly predicted samples is plotted, where a normalized area under the curve (nAUC) reaches 0.81. (B) Scatterplot graphs showing Transcriptomic Angle against actigraphy variables including waketime, most active start time (M10), bedtime, and amplitude; salivary dim light melatonin onset (DLMO); and Morningness–Eveningness Questionnaire (MEQ) score, where a high score indicates a morning chronotype. Correlation *r* and *p* values are shown for partial correlation analyses adjusted for age and sex. Corresponding Bland–Altman (B–A) plots are shown to the right of each scatterplot, where outputs were converted to *z*-scores for calculation of averages and differences. The mean difference in measurements is plotted with 95% confidence limits in dashed red lines.

Forty participants (71.2 ± 4.2 years) were included (Table 1) and stratified by MEQ score. As expected, waketime, bedtime, M10, and DLMO were earlier for “morning type” participants. To evaluate TS accuracy, we compared the TS-derived Transcriptomic Time to the True Time of blood sampling. The normalized area under the curve (nAUC) of the receiver operator characteristic curve reached 0.81 (Figure 1A), consistent with previously published data from young adults [4]. A significant correlation between intraindividual AM and PM Transcriptomic Angles (*r* = 0.797, *p* < 0.001) was also observed, demonstrating internal consistency of the measurements (data not shown).

**Table 1.** Participant demographics and clinical characteristics stratified by Morningness–Eveningness Questionnaire (MEQ) chronotype

Variables	Total	Morning type	Intermediate type
Number (*n*)	40	28	12
Demographics			
Age (y)	71.2 ± 4.2	71.1 ± 4.6	71.3 ± 3.3
Female sex (%)	21 (53%)	17 (60%)	4 (33%)
Caucasian race (%)	38 (95%)	26 (93%)	12 (100%)
Body mass index (kg/m^2^)	29.1 ± 6.7	28.9 ± 7.0	29.5 ± 6.1
Circadian measures, nonactigraphic			
Dim Light Melatonin Onset (time ± min)	08:37 pm ± 92	08:10 pm ± 77*	09:42 pm ± 99*
Total MEQ score	64.4 ± 10.1	69.7 ± 6.3*	52.0 ± 5.1*
Transcriptomic Angle (hours)	2.05 ± 1.72	2.69 ± 1.38*	0.56 ± 1.52*
Actigraphy circadian measures			
M10 (time ± min)	08:19 am ± 108	07:51 am ± 110*	09:22 am ± 71*
Amplitude	279 ± 114	276 ± 114	287 ± 118
Interdaily stability	0.58 ± 0.14	0.59 ± 0.14	0.58 ± 0.14
Intradaily variability	0.83 ± 0.22	0.85 ± 0.24	0.78 ± 0.18
Actigraphy sleep measures			
Bedtime (time ± min)	10:45 pm ± 68	10:20 pm ± 53*	11:42 pm ± 65*
Midsleep (time ± min)	04:10 am ± 27	04:07 am ± 24	04:18 am ± 33
Waketime (time ± min)	07:05 am ± 76	06:33 am ± 63*	08:18 am ± 49*
Total sleep time (hh:mm ± min)	06:43 ± 54	06:40 ± 51	06:47 ± 63
Sleep efficiency (%)	81 ± 8	81 ± 7	80 ± 12
Wake time after sleep onset (min)	60 ± 24	58 ± 19	65 ± 32

All continuous variables are shown as mean *+*standard deviation. M10, start time of most active 10 h.

*p < 0.05 by Student’s *t*-test or chi-squared test comparing morning versus intermediate type.

We hypothesized that individuals with a positive Transcriptomic Angle would have an advanced circadian phase (morning type). Accordingly, Transcriptomic Angle negatively correlated with actigraphically assessed wake time, M10, and bedtime (Figure 1B). A negative correlation was also observed between Transcriptomic Angle and DLMO, the “gold-standard” measure of circadian phase, and with subjective chronotype, as assessed by the MEQ. By contrast, other sleep-related variables and measures of circadian amplitude or fragmentation were not correlated with Transcriptomic Angle (Figure 1B and data not shown). Bland–Altman plots demonstrate general agreement between Transcriptomic Angle and standard measurement techniques (Figure 1B).

In this study, we demonstrate TS accuracy in older adults without the need for algorithm retraining. Moreover, we found that TS-derived Transcriptomic Angle correlates with 3 separate measures of circadian phase including actigraphy, DLMO, and MEQ. Therefore, TS output may allow for transcriptomic circadian phenotyping, which would be useful for clinical and neurological research applications. For example, the delayed circadian phase has been linked to dementia and Alzheimer disease pathology [9, 10], but accurate chronotyping is needed in longitudinal cohort studies to further examine this relationship.

Future work will focus on TS optimization for the assessment of circadian amplitude and fragmentation, and a more diverse cohort will be needed for generalizability. We note the lack of “evening types” in our cohort. However, evening chronotypes are rare among older adults, and the accuracy of TS trained with data from young adults (who tend to have later chronotypes) suggests that TS would perform similarly [4]. Overall, the increasing use of circadian transcriptomics approaches to measure physiological states points towards precision medicine as a tangible reality in the future.
